# Data on a synthetic farm population of the German federal state of North Rhine-Westphalia

**DOI:** 10.1016/j.dib.2021.107007

**Published:** 2021-03-27

**Authors:** Christoph Pahmeyer, David Schäfer, Till Kuhn, Wolfgang Britz

**Affiliations:** a Institute for Food and Resource Economics, University of Bonn, D-53115 Bonn, Germany; b Bioeconomy Science Center (BioSC), Forschungszentrum Jülich, D-52428 Jülich, Germany

**Keywords:** Synthetic farm population, Farm typology, Germany, North Rhine-Westphalia, Farm modeling, Agent-based modeling

## Abstract

Farm-scale and agent-based models draw typically on detailed and preferably spatially explicit single farm data. Data protection standards however restrict or exclude their access, as for example in Germany. We provide data on a synthetic farm population of the German federal state of North Rhine-Westphalia, mainly based on the German Farm Structure Survey 2016 and plot specific crop data from 2019/2020. The population is derived from farm typology at administrative unit level to which the observed plots are allocated afterwards. The data contains 25,858 farms and covers 1.3 million ha of agricultural land, provided at plot scale in a geospatial vector and at farm scale in tabular format. For each plot, the managing farm (including the estimated farm's location), the number of livestock, the cultivated crop, as well as the corresponding administration units are indicated. Furthermore, spatial data such as yield information, soil characteristics, as well as monitoring data on environmental status are attached. The provided data allows for diverse analysis on the farm population in the federal state of North Rhine-Westphalia with farm, agent-based or different bio-physical models. Furthermore, it can serve as a test data set for models which require detailed and spatially explicit farm data.

## Specifications Table

**Table:** 

Subject	Agricultural Economics
Specific subject area	Bio-economic modeling using farm and agent-based models
Type of data	Table Map
How data were acquired	The data was acquired by combining different agricultural farm-level surveys as secondary data sources and spatial data on farmland characteristics.
Data format	Analyzed
Parameters for data collection	Providing and analysing secondary data (1) From farm structure survey, number of farms in different size classes and farm types, agricultural land use in hectares, livestock numbers; both at county and commune level for the year 2016. (2) From the Integrated Administration and Control System (IACS/INVEKOS) agricultural land use for the year 2020. (3) Numerous parameters linked to spatial data (Table 3).
Description of data collection	The underlying secondary data are extracted from open access excel files, literature and from open access spatial data in shape file format.
Data source location	All input and final data set cover the German federal state of North Rhine-Westphalia All used data sources are listed in Table 3.
Data accessibility	The farm typology is available at Mendeley Data at farm and plot scale: https://doi.org/10.17632/75wngh8x4j.1

## Value of the Data

• We provide spatial explicit data of an entire farm population. This is useful for studies considering farm heterogeneity, neighboring effects, and population-wide analysis.
• The spatial explicit single farm data records are especially valuable for the use in farm- and agent-based models.
• The farm population can feed into different types of assessment at farm-scale such as of policy impacts or technology adaption.
• The provided farm population can be used as a test data set for models requiring detailed and spatial explicit farm-level data.
• The data is not only of value for economic modeling exercises, but also for scientific work in fields such as landscape ecology or regional development where spatial explicit farm data are needed.
• The methodology to create the farm population can be transferred to other regions where access to individual farm data records is restricted.

## 1 Data Description

The data set provides a synthetic farm population with single farm data of the German federal state of North Rhine-Westphalia, derived by combining different secondary data sources. This is particularly useful for single farm and agent-based models (ABM) that often require spatially explicit and highly detailed single farm data. The resulting population covers 25,858 single farms and 1.3 million ha of agricultural land in the state. This corresponds to approximately 77% of all farms and 89% of all agricultural land. The state covers a diverse farm population, comprising approximately 8600 specialized arable farms, 4800 specialized pig farms, 9500 specialized cattle farms, and 3100 mixed farms, of varying sizes. They are distributed over different landscapes, such as fertile plains dominated by specialized arable farms, sandy plains with are large share of intensive animal production, and low-mountain ranges characterized by permanent grassland and cattle production. For every farm, estimations of its location, of its managed plots with observed crops and of its livestock numbers are provided. Single farm data at this level of detail is required for spatial explicit or population-wide analysis. However, it is usually not available in Germany due to data protection guidelines. If access is granted, publication of results is restricted, and the handling of the data is governed by complex rules. The synthetic population presented here provides an alternative which reflects key characteristics of the actual farm population without drawing on detailed single farm, data protected information. The provided farms, including their location, do not correspond to observed real-world farms. Instead, they reflect the distribution of key characteristics in the true population and correspond, in their entirety, to observed statistical measurements. All underlying data sources are published and publicly available.

Different data sources are combined to derive the presented population (Table 3). The core sources are a farm typology from Kuhn & Schäfer [1] and frequency tables of farms at commune level (LAU - Local Administrative Units), both based on the German Farm Structure Survey 2016. It is complemented by spatial explicit land use for the crop year 2019/2020, taken from the Integrated Administration and Control System (IACS) for the direct payments of the EU Common Agricultural policy. This land use data is linked to further spatial data such as yield information, soil characteristics, or monitoring data on environmental status.

The derived farm population is supplied at two scales and data formats at a Mendeley repository (http://dx.doi.org/10.17632/75wngh8x4j.1). First, single farm data for the population is provided in CSV format, with one row per farm. The variables, reported in the columns (Table 1), cover a unique farm ID, administrative units, longitude and latitude of the hypothetical farm location, livestock numbers, land use, information on plot size and plot-farmstead-distance, and a list of the managed plots. Second, data for each plot is provided in Shapefile format, reporting its exact spatial location as a polygon. The related attribute table contains additional information per plot (Table 2), covering among others plot and farm ID, plot size, cultivated crop, administrative units, soil parameters, environmental parameters, and regional crop yields. Linkage of the data set can draw on the unique farm ID provided for every plot in the shapefile, or the list of plot IDs reported for each farm in the CSV file.

**Table 1:** Variables of data at farm-level (file *Farm_Population_NRW_farm_data*).

Variable	Type	Description
farmId	Nominal	Unique ID for each farm
farmTypeCategory	Nominal	Groups farms according to their main farming activities which are defined based on the relative contribution of farming activities to the standard output following the EU typology [2]
scr	Nominal	Soil-climate-region of the location of the farmstead
NAME	Nominal	LAU name of the location of the farmstead
LAU	Nominal	LAU code of the location of the farmstead
nuts3	Nominal	NUTS 3 code of the location of the farmstead
lng	Continuous	Longitude of the location of the farmstead
lat	Continuous	Latitude of the location of the farmstead
cows	Continuous	Number of cows [heads]
bulls	Continuous	Number of bulls^1^ [heads]
pigs	Continuous	Number of pigs [heads]
sows	Continuous	Number of sows [heads]
farmSize	Continuous	Total farmland endowment [ha]
arableLand	Continuous	Arable land endowment [ha]
grassLand	Continuous	Permanent Grassland endowment [ha]
Wheats	Continuous	Cereal cultivation area [ha]
RootCrops	Continuous	Root crops cultivation area [ha]
ArableFodder	Continuous	Arable fodder cultivation area [ha]
Oilseeds	Continuous	Oilseeds cultivation area [ha]
ProteinCrops	Continuous	Protein crops cultivation area [ha]
OrnamentalPlants	Continuous	Ornamental plants cultivation area [ha]
EnergyCrops	Continuous	Energy crops cultivation [ha]
avgPlotSize	Continuous	Average plot size of farm [ha]
medianPlotSize	Continuous	Median plot size of farm [ha]
deviationPlotSize	Continuous	Standard deviation of plot sizes of farm [ha]
avgPlotDistance	Continuous	Average plot-farmstead distance of farm [km]
medianPlotDistance	Continuous	Median plot-farmstead distance of farm [km]
deviationPlotDistance	Continuous	Deviation from average plot-farmstead distance of farm [km]
plots	Nominal	List of unique plot IDs assigned to the farm, separated by semi-colons

1 Number of bulls calculated based on livestock units provided by Kuhn & Schaefer [1]. *Abbreviations:* LAU - Local Administrative Units, NUTS - Nomenclature of Territorial Units for Statistics.

**Table 2:** Variables of data at plot-level (file Farm_Population_NRW_plot_data).

Variable	Type	Description
id	Nominal	Unique plot ID
farmId	Nominal	Unique farm ID of the farm managing the plot
plotSize	Continuous	Size [ha]
Distance	Continuous	Plot-farmstead distance [km]
FLIK	Nominal	Unique field block ID
cultivation	Nominal	Crop cultivated
NUTS1	Nominal	Federal state
NUTS2	Nominal	Administrative district
NUTS3	Nominal	County
LAU	Nominal	LAU code
NAME	Nominal	LAU name
N-content soil	Continuous	Total N in soil [g kg^−1^]
P-content soil	Continuous	Total P in soil [g kg^−1^]
K-content soil	Continuous	Extractable K in soil [g kg-1]
LC0_Desc	Nominal	Description of the most proximate location where N-/P-/K data was obtained from
Bodenzahl	Continuous	Soil value (German: Bodenwertzahl)
SQR	Continuous	Müncheberg soil quality rating
soilType	Nominal	Soil type
soilCode	Nominal	Soil type code
humus	Ordinal	Humus content [%]
redArea	Nominal	Plot in red zone (nitrate pollution hotspot according to DVO 2020)
eutrophicArea	Nominal	Plot in eutrophic area (phosphate pollution hotspot according to DVO 2020)
water_erosion_code	Discrete	Water erosion risk classes (0 = no risk, 1 = medium risk, 2 = high risk)
water_erosion_lvl	Ordinal	Water erosion risk classes in textual form (German)
wind_erosion	Nominal	High risk of wind erosion
slopeGradient	Ordinal	Slope gradient class [%]
WHG	Nominal	Affected according to §38a WHG (with distance indication)^1^
DVO	Nominal	Affected according to fertilization regulation §5 (with distance indication)^1^
slopeBufferMarginArea	Continuous	Area of the plot that is affected by either WHG and / or DVO [ha]^1^
slopeBufferMarginPoly	–	A GeoJSON polygon outlining the part of the plot affected by the slope gradient scenery 1
sugarbeets_yield	Continuous	Average sugar beet yield at NUTS 3 level [dt ha^−1^]
oats_yield	Continuous	Average oat yield at NUTS 3 level [dt ha^−1^]
summer_barley_yield	Continuous	Average summer barley yield at NUTS 3 level [dt ha^−1^]
triticale_yield	Continuous	Average triticale yield at NUTS 3 level [dt ha^−1^]
winter_wheat_yield	Continuous	Average winter wheat yield at NUTS 3 level [dt ha^−1^]
potatoes_yield	Continuous	Average potato yield at NUTS 3 level [dt ha^−1^]
corn_silage_yield	Continuous	Average corn silage yield at NUTS 3 level [dt ha^−1^]
winter_rye_yield	Continuous	Average winter rye yield at NUTS 3 level [dt ha^−1^]
rapeseed_yield	Continuous	Average rapeseed yield at NUTS 3 level [dt ha^−1^]
winter_barley_yield	Continuous	Average winter barley yield at NUTS 3 level [dt ha^−1^]

1 Variable not presented if not applicable for the plot. *Abbreviations:* DVO – German Fertilization Ordinance, K - potassium, LAU - Local Administrative Units, N - nitrogen, NUTS - Nomenclature of Territorial Units for Statistics, P - phophorus, WHG - German Federal Water Act.

## 2 Materials and Methods

**Fig. 1:**
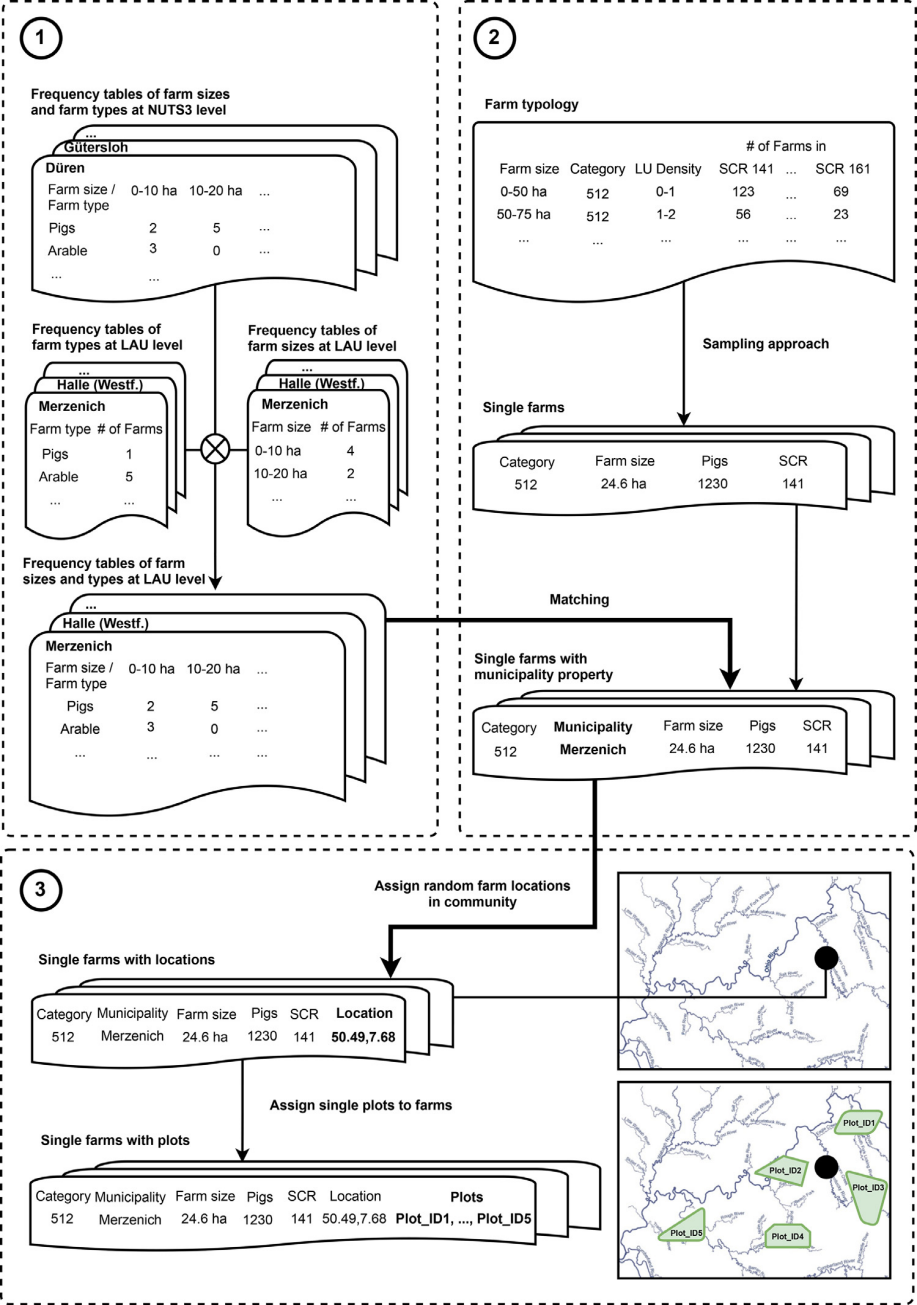
Overview on the synthetic farm population generation for the German federal state of North-Rhine Westphalia.

In the following, we document the methodology for creating the synthetic farm population for the German federal state of North Rhine-Westphalia. The population generation can be understood as descriptive research as we aim at characterizing and depicting the farm population in North Rhine-Westphalia without testing any hypothesis or drawing conclusions. It consists of three major steps as outlined in Fig. 1. First, we generate farm frequency tables at LAU level. Second, we process the contingency tables sourced from the farm typology by Kuhn and Schäfer [1], match it to a fitting farm from the previously generated frequency tables at LAU level, and create a spatially implicit farm population. Third, the farms are assigned random locations within the boundaries of their LAU. Finally, the observed plots, sourced from the publicly available IACS dataset, are assigned to the individual farms, based on their aspired farm size, crop cultivation specialization, and grassland endowment. The latter steps then turn the spatially implicit into a spatially explicit farm population. The created synthetic farm population is linked to further spatial data on yields, soil characteristics, and monitoring data on environmental status.

The data sources used in our methodology are outlined in Table 3. The creation of the synthetic population draws on two sources, the German Farm Structure Survey and the IACS data on land use. The Farm Structure Survey, carried out every three to four years, provides single farm data for all farms above a certain size threshold. It is the basis for the typology by Kuhn and Schäfer [1] as well as the official farm statistics which are used to create the frequency tables. The typology contains only the most important farm types and, therefore, the estimated population does cover neither all farms nor all agricultural land in North Rhine-Westphalia (see Kuhn and Schäfer [1] for details). IACS data on land use for each plot are reported annually by the farmers to determine direct payments from the EU Common Agricultural Policy. The agency collecting the data offers public access to them, however without information on the farmer managing the plot, and aggregating single crop information mostly to group of crops. As the data are spatially explicit, further spatial data relevant for agricultural land use can be attached as reported in Table 3. Note that except for the farm typology created by Kuhn and Schäfer [1], most dataset are readily available in other German federal states as well.

**Table 3:** Data sources.

Name	Description	Source of data
*Sources for population creation*		
Farm typology by Kuhn & Schäfer [1]	A farm typology for the German federal state of North Rhine-Westphalia based on the Farm Structure Survey 2016	http://www.ilr.uni-bonn.de/agpo/publ/dispap/download/dispap18_01.pdf
Integrated Administration and Control System (IACS/INVEKOS) data 2019/2020	Declared and verified eligible parcels for the EU funding, open data from the Chamber of Agriculture North Rhine-Westphalia	https://www.opengeodata.nrw.de/produkte/umwelt_klima/bodennutzung/landwirtschaft/
Official farm statistics from IT NRW 2018	Official farm statistics on LAU and NUTS 3 levels based on the Farm Structure Survey 2016	https://www.it.nrw/statistik/wirtschaft-und-umwelt/land-und-forstwirtschaft/struktur-der-landwirtschaftlichen-betriebe
*Spatial data linked to plots*		
Administrative location units (variables NUTS 1, NUTS 2, NUTS 3, LAU)	Spatial data on administrative location units at NUTS 1, NUTS 2, NUTS 3 and LAU level	https://github.com/eurostat/Nuts2json https://www.opengeodata.nrw.de/produkte/geobasis/vkg/dvg/dvg1/
Nutrient content soil (variables N-content soil, P-content soil, K-content soil)	Total N (defined using ISO 11,261:1995 method), total P (defined using ISO 11,263:1194 method), extractable K (defined using USDA−NRCS, 2004 method) based on LUCAS 2015 TOPSOIL data	https://esdac.jrc.ec.europa.eu/content/lucas2015-topsoil-data#tabs-0-description=1
Soil value (variable Bodenzahl)	German soil quality ranking between 0 and 100	https://www.geoportal.nrw/suche?lang=de&searchTerm=3E7CC528–6560–4BBE-AAB0–7DE2417EF993
Soil quality rating (variable SQR)	Müncheberg soil quality rating, indicator-based assessment of soil quality and crop yields potential described by Mueller et al. [3]	https://www.bgr.bund.de/DE/Themen/Boden/Ressourcenbewertung/Ertragspotential/Ertragspotential_node.html
Soil type (variables soilType, soilCode)	Soil type and corresponding code based on the classification of the LUFA NRW	https://www.geoportal.nrw/suche?lang=de&searchTerm=3E7CC528–6560–4BBE-AAB0–7DE2417EF993
Humus content of soil (variable humus)	Median content of organic matter in the topsoil, given in percent classes (e.g. ‘1-<2%’)	https://www.bgr.bund.de/DE/Themen/Boden/Informationsgrundlagen/Bodenkundliche_Karten_Datenbanken/Themenkarten/HUMUS1000OB/humus1000ob_inhalt.html
Nitrate and phosphate pollution hot spots (variables redArea, eutrophicArea)	Area defined as nitrate and phosphate pollution hot spots in accordance with $13 DVO	https://www.opengeodata.nrw.de/produkte/umwelt_klima/wasser/duev/
Erosion risk (variables water_erosion_code, water_erosion_lvl, wind_erosion)	Risk classes for water erosion and areas at risk for wind erosion linked agri-environmental measures	https://www.opengeodata.nrw.de/produkte/umwelt_klima/bodennutzung/landwirtschaft/
Slope and distance to surface waters (variables slope gradient, WHG, DVO, slopeBufferMarginArea, slopeBufferMarginPoly)	Sloped areas neighboring surface waters with management restrictions according to WHG §38a and DVO §5	https://www.opengeodata.nrw.de/produkte/umwelt_klima/bodennutzung/landwirtschaft/
Regional yields	Regional yield level for main arable crops based on data from 2014 to 2017	https://flf.julius-kuehn.de/webdienste/webdienste-des-flf/ernteertraege.html

*Abbreviations:* DVO – German Fertilization Ordinance, K - potassium, LAU - Local Administrative Units, N – nitrogen, NUTS - Nomenclature of Territorial Units for Statistics, P – phosphorus, WHG – German Federal Water Act.

### 2.1 Generation of frequency tables at municipality level

This section presents the development of frequency tables which contain the frequencies of different farm types and size classes at the LAU level. The underlying code in the programming language GAMS is provided in a software versioning system.^1^ Official statistics provide frequency tables for farm type and size classes, total utilized agricultural land, and agricultural land differentiated by land use at NUTS 3 and higher level, only. At LAU level, only vectors on the frequency of different farm types and size classes are reported. Our approach estimates probable frequency tables at LAU level, drawing on the frequency tables at NUTS 3 level and the vectors at LAU level. We use solely data from IT NRW 2018 for the estimation (Table 3).

The constraints of the estimation framework relate to adding up conditions at LAU level. Let *x_s,c,t_* denote the unknown number of farms of a certain size class *s* and type of specialization *t* in each of the 396 LAU. *c, d_s,c_* and *d_t,c_* are the given data on the number of farms of a certain size class, respectively, type, and *d_c_* on the total number of farms. The following adding up conditions eq. (1)-(3) should hold for any estimated frequency table of the farming population at LAU level *x_s,c,t_*:

(1) $$\sum_{s}x_{s,c,t}=d_{t,c}$$

(2) $$\sum_{t}x_{s,c,t}=d_{s,c}$$

(3) $$\sum_{s,t}x_{s,c,t}=d_{c}$$

After defining the adding up conditions on LAU level, we apply the same approach on NUTS 3 and 2 level as sometimes cells in frequency tables are left blank due to data protection rules. Let *k* denote county (NUTS 3, Kreis, 29 units) and *r* district (NUTS 2, Regierungsbezirk, 5 units) which are the two administrative units above LAU level where frequency tables on the number of farms by size class and type are available. Taking this additional information into account, we can add the following adding up conditions eq. (4)-(5) from LAU to NUTS 3 and from NUTS 3 to NUTS 2 to the estimation framework.

(4) $$\sum_{c\;\in \;k}x_{s,t,c}=\;d_{s,t,k}=x_{s,t,k}$$

(5) $$\sum_{c\;\in \;r}x_{k,t,c}=\;d_{s,t,r}=x_{s,t,r}$$

The estimation problem is defined as a highest posterior density problem (HPD).^2^ We assume a-priori the relative, but unknown shares of the distribution by size class and type at LAU level are equal to the observed one at NUTS 3 level. Thus, *s* in the objective function (eq. (6)) denotes the shares describing the empirical distribution observed at NUTS 3 level:

(6) $$\min\sum_{s,t,c}\frac{x_{s,t,c}}{d_{c}}-s_{s,t,c}$$

The resulting estimates for *x* are real numbers and not counts, as required for the frequency tables. In order to convert them into a distribution of integers, we introduce bounds around each estimated *x_s,t,c_* representing its floor and ceiling values. We next construct a new estimator where eq. (6) is replaced by an objective function which shifts the value towards an integer. To do that the estimator minimizes the squared difference between the estimates of *x_s,t,c_* and a number which is smaller than its lower bound if it is closer to the lower bound or higher than its upper bound otherwise. Any *x_s,t,c_* which is already an integer is automatically fixed as its floor and ceiling are identical. The additional estimation is repeated several times until all estimates *x* take on integer values

### 2.2 Generation of the farm population

Given the number of farms at LAU level in each category, we start to generate the spatially explicit farm population. The corresponding code, as well as the linkage of spatial data to the farm population is written in the programming language Node.js, and provided in a software versioning repository.^3^

As stated previously, the analysis builds on the North Rhine-Westphalian farm typology published by Kuhn & Schäfer [1]. It differentiates farm types according to (1) type of farming, (2) size class in ha und (3) livestock density in livestock units (LU) per ha in different classes, and reports their numbers at the level of so-called soil-climate-regions (SCRs, generally consists of multiple NUTS 3 regions), which reflect zones of similar farming conditions.

The typology reports for each farm type the number of farms as well as statistics (mean, median, standard deviation) on core farm characteristics, including among others farm size in ha, arable and grassland endowment, and livestock density in LU, in total and for the animal categories pigs, sows, dairy cows, and other cattle. Based on the frequency tables, a sampling approach generates a matching farm population, and attaches characteristics according to the information found in the farm typology to each farm. The information on size and livestock density in the farm typology refers to certain classes (e.g. 0–50 ha, or 0–1 LU), accordingly, a truncated normal distribution is assumed when drawing these characteristics for a hypothetical farm in a cell of the typology.

In order to determine the nonparametric skew (S) of each farm characteristics, we calculate the skew using

$$S=\;\frac{\mu -\nu }{\sigma }$$

where μ is the populations mean, ν the populations median, and σ is the populations standard deviation for the given variable.

Due to data protection, statistics on farm characteristic for some farm types has been blackened in the farm typology. In these cases, average values given the farms size cluster are assumed for the variables with missing distribution data. For instance, farms in a size cluster between 0 ha and 50 ha would be assigned to 25 ha. Detailed comments in the relevant code sections report further assumptions made in the sampling approach. Following this sampling approach, a spatially implicit farm population containing a list of farms with specific values for each farm characteristic is generated (Fig. 1).

So far, the location of a farm can only be assigned at SCR level. To advance here, the farm frequency tables at LAU level described in the Section 2.1 are used. For each farm in the farm population, a farm at LAU level is matched, considering a) its farm type, b) its size class (e.g. 10–30 ha) and c) that the LAU falls in the SCR of the farm. Once a matching farm from the frequency tables is found, the LAU property from the match is added to the respective farm of the farm population. Thereby, the number of farms in the frequency table of this LAU that are not yet distributed is decreased.. The LAU frequency tables and the farm typology are not completely harmonized, even if they stem originally from the same raw data source. Therefore, few farms cannot be matched, as each farm in the frequency table is at SCR level only used once in the matching procedure. In these cases, a farm is chosen for which solely the SCR and size cluster matches.

### 2.3 Linking spatial data to the farm population

Once each farm is assigned to a LAU, specific farm locations are designated to the farms of the generated farm population. Farm locations are assumed to be a random vertex from an arbitrarily chosen field (polygon) within the boundaries of the LAU the farm belongs to. If more than 50% of the farm's land endowment is arable land, only arable plots are considered for the farm location. Respectively, if more than 50% of the farms land endowment is grassland, only grassland plots are considered. If a vertex has already been defined as a farm location, the algorithm is recursively called until a new, unused farm location is found.

In a final stage of the generation of the spatially explicit farm population, individual plots are assigned to the farms. The plots in the federal state are shuffled to guarantee a random order, and afterwards evaluated for their suitability for a given farm. The assignment procedure generally differentiates between grassland and arable plots.

For grassland plots, farms that are within a 30 km driving radius around the plot are filtered and sorted by distance in ascending order. To maximize the efficiency of the algorithm, filtering of the farms is done using a spatial index based on a flat k-d tree as proposed by Bentley [5]. Subsequent to the farm filtering, the closest farm is searched for where the sum of the current plot and the current grassland endowment of the farm does not exceed the aspired grassland endowment of the farm (including a buffer of 5%). Also, a check is incorporated prohibiting a farm to exceed its farm size cluster. If a matching farm is found, the current plot is added to the farm, otherwise the plot is added to a list of unused plots.

For arable plots, farms within close proximity (5 km) are filtered and sorted based on their suitability for the given crop cultivated on the current plot. This is done to increase the probability of e.g. a specialized cereal producing farm to obtain plots cultivated with cereals. Again, a farm is searched for where the sum of the current plot and the current arable land endowment of the farm does not exceed the aspired arable land endowment of the farm (including a buffer of 5%). In addition to the check prohibiting the farm to exceed its farm size cluster, another check is incorporated prohibiting farms above 10 ha (the threshold where the EU Greening obligation becomes binding) to exceed certain crop shares. In case no suitable farm is found within 5 km for the given plot, the radius is increased to 30 km. Also, the sorting is solely based on the farm to field distance. If a matching farm is found, the current plot is added to the farm, otherwise the plot is added to the list of unused plots.

Farms that are within 95% of their aspired farm size are labelled as finished and removed from the list of farms considered in the evaluation of the following plots. After the first round of the assignment procedure, the list of unused plots is iterated over again.

In this second round, the plots are assigned to the most proximate farm as long as adding the plot to the farm does not exceed the farms overall aspired farm size. Here, the aspired arable and grassland properties are ignored, allowing for a deviation of these values in case no sufficient arable or grassland is available in the region of the farm.

Using the approach outlined in this section, matching the approx. 700.000 plots and 25.500 farms in the federal state of North Rhine-Westphalia takes less than 10 min.

## CRediT Author Statement

**Christoph Pahmeyer:** Conceptualization, Supervision, Methodology Chapter 2.2 & 2.3, Software Chapter 2.2 & 2.3**,** Writing-Chapter 2.2 & 2.3, Writing-Reviewing and Editing; **David Schäfer:** Methodology Chapter 2.1, Software Chapter 2.1, Writing-Chapter 2.1, Writing-Reviewing and Editing; **Till Kuhn:** Conceptualization, Supervision, Writing-Original draft preparation, Writing Chapter 1, Writing-Reviewing and Editing; **Wolfgang Britz:** Methodology Chapter 2.1 & 2.3, Software Chapter 2.1, Writing-Reviewing and Editing.

## Declaration of Competing Interest

The authors declare that they have no known competing financial interests or personal relationships which have or could be perceived to have influenced the work reported in this article.

## Footnotes

1 The code is hosted at the following Github repository: https://doi.org/10.5281/zenodo.4633234.

2 For further discussion on HPD estimators please refer to Heckelei et al. [4].

3 The code is hosted at the following Github repository: https://doi.org/10.5281/zenodo.4668581.
